# Supplementary material for: A decrease in reports on road-killed animals based on citizen science during COVID-19 lockdown
Source: PeerJ. 2021 Nov 23;9:e12464. doi: 10.7717/peerj.12464 (PMC8621783; doi:10.7717/peerj.12464)
Supplement: Supplemental Information 1 [file peerj-09-12464-s001.pdf]

Projekt Roadkill während Corona

Sehr geehrte Teilnehmer*in,

vielen Dank, dass Sie sich 3 Minuten Zeit nehmen um die folgenden Fragen bezüglich der Teilnahme am Projekt Roadkill während der Corona Krise in Österreich von 16. März 2020 bis 13. April 2020 zu beantworten. Ihre Antworten helfen uns dabei die Meldungen, die während dieser Zeit bei uns eingegangen sind, besser einordnen zu können.

In dieser Umfrage stellen wir keine Fragen zu Ihrer Person, daher ist die Umfrage vollkommen anonym und es kann nicht auf Ihre Person zurück geschlossen werden.

Sollten Sie Fragen zu der Umfrage haben, schreiben Sie uns bitte an office@roadkill.at

In dieser Umfrage sind 16 Fragen enthalten.

Die Umfrage:

Melden Sie schon länger als ein Jahr Roadkills im Projekt Roadkill? *

Bitte wählen Sie nur eine der folgenden Antworten aus:

- ○ Ja
- ○ Nein

Hat sich die Zahl der von Ihnen gemeldeten Roadkills im Zeitraum vom 16.3.2020 bis 13.4.2020 gefühlt verändert? *

❶ Bitte wählen Sie eine der folgenden Antworten:
Bitte wählen Sie nur eine der folgenden Antworten aus:

- ○ Ich habe gefühlt mehr Roadkills gemeldet.
- ○ Ich habe gefühlt weniger Roadkills gemeldet.
- ○ Ich habe gefühlt gleich viele Roadkills gemeldet.

Hat sich im Zeitraum vom 16.3.2020 bis 13.4.2020 die Länge Ihrer Wege, von denen Sie potentiell Roadkills melden, geändert? *

Bitte wählen Sie nur eine der folgenden Antworten aus:

- ○ Ja
- ○ Nein

Hat sich die Länge der Wege reduziert oder erhöht? *

Beantworten Sie diese Frage nur, wenn folgende Bedingungen erfüllt sind:
Antwort war 'Ja' bei Frage '3 [F3]' (Hat sich im Zeitraum vom 16.3.2020 bis 13.4.2020 die Länge Ihrer Wege, von denen Sie potentiell Roadkills melden, geändert?)

❶ Bitte wählen Sie eine der folgenden Antworten:
Bitte wählen Sie nur eine der folgenden Antworten aus:

- ○ Reduziert
- ○ Erhöht

Um wie viel hat sich die Länge der Wege gefühlt reduziert? *

Beantworten Sie diese Frage nur, wenn folgende Bedingungen erfüllt sind:
Antwort war 'Reduziert' bei Frage '4 [F31]' (Hat sich die Länge der Wege reduziert oder erhöht?)

❶ Bitte wählen Sie eine der folgenden Antworten:
Bitte wählen Sie nur eine der folgenden Antworten aus:

- ○ 0-25%
- ○ 26-50%
- ○ 51-75%
- ○ 76-100%

Um wie viel hat sich die Länge der Wege gefühlt erhöht? *

Beantworten Sie diese Frage nur, wenn folgende Bedingungen erfüllt sind:
Antwort war 'Erhöht' bei Frage '4 [F31]' (Hat sich die Länge der Wege reduziert oder erhöht?)

❶ Bitte wählen Sie eine der folgenden Antworten:
Bitte wählen Sie nur eine der folgenden Antworten aus:

- ○ 0-25%
- ○ 26-50%
- ○ 51-75%
- ○ 76-100%
- ○ mehr als 100%

Hat sich im Zeitraum vom 16.3.2020 bis 13.4.2020 die Häufigkeit, mit der Sie auf Ihren Wegen unterwegs waren, geändert? *

Bitte wählen Sie nur eine der folgenden Antworten aus:

- ○ Ja
- ○ Nein

Hat sich die Häufigkeit, mit der Sie auf Ihren Wegen unterwegs waren, reduziert oder erhöht? *

Beantworten Sie diese Frage nur, wenn folgende Bedingungen erfüllt sind:
Antwort war 'Ja' bei Frage '7 [F4]' (Hat sich im Zeitraum vom 16.3.2020 bis 13.4.2020 die Häufigkeit, mit der Sie auf Ihren Wegen unterwegs waren, geändert?)

❶ Bitte wählen Sie eine der folgenden Antworten:
Bitte wählen Sie nur eine der folgenden Antworten aus:

- ○ Reduziert
- ○ Erhöht

Um wie viel hat sich die Häufigkeit, mit der Sie auf Ihren Wegen unterwegs waren, gefühlt reduziert? *

Beantworten Sie diese Frage nur, wenn folgende Bedingungen erfüllt sind:
Antwort war 'Reduziert' bei Frage '8 [F41]' (Hat sich die Häufigkeit, mit der Sie auf Ihren Wegen unterwegs waren, reduziert oder erhöht?)

❶ Bitte wählen Sie eine der folgenden Antworten:
Bitte wählen Sie nur eine der folgenden Antworten aus:

- ○ 0-25%
- ○ 26-50%
- ○ 51-75%
- ○ 76-100%

Um wie viel hat sich die Häufigkeit, mit der Sie auf Ihren Wegen unterwegs waren, gefühlt erhöht? *

Beantworten Sie diese Frage nur, wenn folgende Bedingungen erfüllt sind:
Antwort war 'Erhöht' bei Frage '8 [F41]' (Hat sich die Häufigkeit, mit der Sie auf Ihren Wegen unterwegs waren, reduziert oder erhöht?)

❶ Bitte wählen Sie eine der folgenden Antworten:
Bitte wählen Sie nur eine der folgenden Antworten aus:

- ○ 0-25%
- ○ 26-50%
- ○ 51-75%
- ○ 76-100%
- ○ mehr als 100%

Hat sich im Zeitraum vom 16.3.2020 bis 13.4.2020 die Wegführung, von denen Sie potentiell Roadkills melden, geändert? *

Bitte wählen Sie nur eine der folgenden Antworten aus:

- ○ Ja
- ○ Nein

Wie hat sich die Wegführung, von der Sie potentiell Roadkills melden, geändert?
*

Beantworten Sie diese Frage nur, wenn folgende Bedingungen erfüllt sind:
Antwort war 'Ja' bei Frage '11 [F5]' (Hat sich im Zeitraum vom 16.3.2020 bis 13.4.2020 die Wegführung, von denen Sie potentiell Roadkills melden, geändert?)

Bitte wählen Sie die zutreffende Antwort für jeden Punkt aus:

	von großteils ...			zu großteils ...		
	**besiedeltes Gebiet (z.B. Dörfer, Städte)**	**landwirtschaftlich genutztes Gebiet (z.B. Felder, Äcker, Obst- bzw. Weinbaugebiete)**	**forstwirtschaftlich genutztes Gebiet (z.B. Wälder)**	**besiedeltes Gebiet (z.B. Dörfer, Städte)**	**landwirtschaftlich genutztes Gebiet (z.B. Felder, Äcker, Obst- bzw. Weinbaugebiete)**	**forstwirtschaftlich genutztes Gebiet (z.B. Wälder)**
**Wegführung**	○	○	○	○	○	○

Hat sich im Zeitraum vom 16.3.2020 bis 13.4.2020 die Art der Straßen, auf denen Sie großteils unterwegs sind, im Vergleich zu früher geändert? *

Bitte wählen Sie nur eine der folgenden Antworten aus:

- ○ Ja
- ○ Nein

Wie hat sich die Art der Straßen, auf denen Sie großteils unterwegs sind, im Vergleich zu früher geändert?
*

Beantworten Sie diese Frage nur, wenn folgende Bedingungen erfüllt sind:
Antwort war 'Ja' bei Frage '13 [F6]' (Hat sich im Zeitraum vom 16.3.2020 bis 13.4.2020 die Art der Straßen, auf denen Sie großteils unterwegs sind, im Vergleich zu früher geändert?)

Bitte wählen Sie die zutreffende Antwort für jeden Punkt aus:

	von großteils ...				zu großteils ...			
	**Forststraße oder Feldweg (nicht asphaltiert, außerhalb von Ortgebieten)**	**innerorts (im Ortsgebiet, Geschwindigkeitsbegrenzung bis 50km/h)**	**Überlandstraßen (zwischen Gemeinden, Geschwindigkeitsbegrenzung 70-100km/h)**	**Schnellstraßen/Autobahnen (mehrspurig, Geschwindigkeitsbegrenzung >100km/h)**	**Forststraße oder Feldweg (nicht asphaltiert, außerhalb von Ortgebieten)**	**innerorts (im Ortsgebiet, Geschwindigkeitsbegrenzung bis 50km/h)**	**Überlandstraßen (zwischen Gemeinden, Geschwindigkeitsbegrenzung 70-100km/h)**	**Schnellstraßen/Autobahnen (mehrspurig, Geschwindigkeitsbegrenzung >100km/h)**
**Art der Straße**	○	○	○	○	○	○	○	○

Hat sich im Zeitraum vom 16.3.2020 bis 13.4.2020 die Art der Fortbewegung im Vergleich zu früher geändert? *

Bitte wählen Sie nur eine der folgenden Antworten aus:

- ○ Ja
- ○ Nein

Wie hat sich die Art der Fortbewegung geändert?
*

Beantworten Sie diese Frage nur, wenn folgende Bedingungen erfüllt sind:
Antwort war 'Ja' bei Frage '15 [F7]' (Hat sich im Zeitraum vom 16.3.2020 bis 13.4.2020 die Art der Fortbewegung im Vergleich zu früher geändert?)

Bitte wählen Sie die zutreffende Antwort für jeden Punkt aus:

	von großteils ...						zu großteils ...					
	**zu Fuß**	**Fahrrad**	**Motorrad**	**PKW**	**LKW**	**anders (z.B. öffentliche Verkehrsmittel)**	**zu Fuß**	**Fahrrad**	**Motorrad**	**PKW**	**LKW**	**anders (z.B. öffentliche Verkehrsmittel)**
**Fortbewegungsart**	○	○	○	○	○	○	○	○	○	○	○	○

Vielen Dank für Ihre Zeit!

Die Ergebnisse der Umfrage werden auf unserer Projekt-Website veröffentlicht.

Bei Fragen wenden Sie sich bitte an office@roadkill.at

Ansonsten freuen wir uns Sie bald wieder auf unserer Projekt-Website begrüßen zu dürfen.

04.05.2020 – 00:00

Übermittlung Ihres ausgefüllten Fragebogens:
Vielen Dank für die Beantwortung des Fragebogens.
