# Supplementary material for: A decrease in reports on road-killed animals based on citizen science during COVID-19 lockdown
Source: PeerJ. 2021 Nov 23;9:e12464. doi: 10.7717/peerj.12464 (PMC8621783; doi:10.7717/peerj.12464)
Supplement: Supplemental Information 3 [file peerj-09-12464-s003.docx]

**Annex**

Survey questions in English. The respective question code from Table 2 is shown in parenthesis.

- Question 1 (F1): Have you been reporting roadkills in Project Roadkill for more than a year?
  - - - Possible answers: Yes/No
- Question 2 (F2): Did the number of roadkills you reported change in the period from 16.3.2020 to 13.4.2020?
  - - - Possible answers: I've reported more roadkills in my perception/I've reported fewer roadkills in my perception/nothing has changed in my perception
- Question 3 (F3): Has the length of your routes from which you potentially report roadkills changed in the period from 16.3.2020 to 13.4.2020?
  - - - Possible answers: Yes/No
  - Subquestion 3.1 (F31): Has the length of the paths been reduced or increased?
    - - Possible answer: Reduced
        - Subquestion 3.1.1 (F311): By how much did the length of the paths feel reduced?

Possible answers: 0-25%/26-50%/51-75%/76-100%

- - - - Possible answer: Increased
        - Subquestion 3.1.2 (F312): By how much did the length of the paths feel increased?

Possible answers: 0-25%/26-50%/51-75%/76-100%

- Question 4 (F4): In the period from 16.3.2020 to 13.4.2020 did the frequency with which you travelled on your routes change?
  - - - Possible answers: Yes/No
  - Subquestion 4.1 (F41): Has the frequency with which you travelled on your routes been reduced or increased?
    - - Possible answer: Reduced
        - Subquestion 4.1.1 (F411): By how much did you feel the frequency with which you travelled on your paths was reduced?

Possible answers: 0-25%/26-50%/51-75%/76-100%

- - - - Possible answer: Increased
        - Subquestion 4.1.2 (F412): By how much did you feel the frequency with which you travelled on your paths was increased?

Possible answers: 0-25%/26-50%/51-75%/76-100%

- Question 5 (F5): In the period from 16.3.2020 to 13.4.2020, has the route from which you potentially report roadkills changed?
  - - - Possible answers: Yes/No
  - Subquestion 5.1 (F51n): How has it changed?

Possible answers:

From mainly...settled areas (e.g. villages, towns)/agricultural areas (e.g. fields, arable land, orchards and vineyards)/forestry areas (e.g. forests)

To mainly...settled areas (e.g. villages, towns)/agricultural areas (e.g. fields, arable land, orchards and vineyards)/forestry areas (e.g. forests)

- Question 6 (F6): In the period from 16.3.2020 to 13.4.2020, has the type of roads on which you mostly travel changed compared to the past?
  - - - Possible answers: Yes/No
  - Subquestion 6.1 (F61n): How has it changed?

Possible answers:

From mainly... forest road or field path (unpaved, outside of local areas)/in town (in the local area, speed limit up to 50km/h)/Interurban roads (between municipalities, speed limit 70-100km/h)/Highways/motorways (multi-lane, speed limit >100km/h)

To mainly...forest road or field path (unpaved, outside of local areas)/in town (in the local area, speed limit up to 50km/h)/Interurban roads (between municipalities, speed limit 70-100km/h)/Highways/motorways (multi-lane, speed limit >100km/h)

- Question 7 (F7): In the period from 16.3.2020 to 13.4.2020 has the mode of transportation changed compared to the past?
  - - - Possible Answers: Yes/No
  - Subquestion 7.1 (F71): How has it changed?

Possible answers:

From mainly...on foot/by bike/by motorcycle/by car/by truck/other (e.g. public transport)

To mainly...on foot/by bike/by motorcycle/by car/by truck/other (e.g. public transport)
